# Audit on the Quality of Outpatient Letters From Cherrywood Clinic

**DOI:** 10.1192/bjo.2022.423

**Published:** 2022-06-20

**Authors:** Armaan Akhtar, Faisal Badshah

**Affiliations:** 1 Barchester Healthcare, Windermere House, Hull, United Kingdom; 2 Pennine Acute Trust, Oldham, United Kingdom

## Abstract

**Aims:**

Letters between secondary and primary care are an integral part of continuity of patient care. It is crucial letters are comprehensible, focused and useful. The quality of letters can be of a variable standard, we aim to see if the letters sent from Cherrywood clinic are in line with the Royal College guidance.

**Methods:**

Data were collected manually by 2 doctors using dictated clinic letters and patient notes, from the 3 community teams. 20 outpatient letters were sequentially selected from each team from the 1st to 31st of March 2017; 60 letters in total. The letters were divided equally between consultants and junior doctors. In the team where there were 2 Consultants; 5 letters of each were taken, and in the team where there was a junior doctor and a specialist registrar, 5 letters from each were taken. The data were collated onto an Excel spread sheet and analysed.

1. Demographic Details including Name, Date of Birth, Address and the Date of Appointment
2. Who was the patient been seen by; Consultant or Junior doctor (FY/GPST/CT/SPR)
3. Current diagnosis
4. Current medication including doses
5. Mental State Examination (MSE) findings
6. An update of the current problem(s)
7. Current/relevant Risks
8. Plan/recommendations
9. Follow-up plans

**Results:**

Of the Consultant letters the diagnosis, medication and dosage was mentioned in 93%, 93% and 90% respectively. Mental state was found in 66%, risks in 83% and follow-up plans in 96%.

Most of the content derived from the registrar letters were unremarkable; with 80% in MSE in the 5 audited letters.

In the Junior doctor letters; the diagnosis was mentioned in 88% of letters, medication and dosage 76%, mental state 100%, risks 80%, follow-up 100%.

**Conclusion:**

Our letters are largely meeting the Royal College standards, more than 85% of the data were up to the standard. The main area's to improve are;

- Documentation of the MSE.
- The medication and the dosages.
- Diagnosis.
- Risks should always be present.

The areas which require improvement are the areas which are essential for GPs to safely manage psychiatric patients in the community.

